# Hope for the Best, Prepare for the Worst—An Assessment of Flood Preparedness at Primary Health Care Facilities in Central Vietnam

**DOI:** 10.3390/ijerph15122689

**Published:** 2018-11-29

**Authors:** Andreas Älgå, Thi Anh Thu Dang, Dell D. Saulnier, Gia Thanh Nguyen, Johan von Schreeb

**Affiliations:** 1 Centre for Research on Health Care in Disasters, Health Systems and Policy Research Group, Department of Public Health Sciences, Karolinska Institutet, 17177 Stockholm, Sweden; dell.saulnier@ki.se (D.D.S.); johan.von.schreeb@ki.se (J.v.S.); 2 Department of Environmental and Occupational Health, Faculty of Public Health, Hue College of Medicine and Pharmacy, Hue University, Hue City, Vietnam; dtathu@huemed-univ.edu.vn (T.A.T.D.); gianguyen175@gmail.com (G.T.N.)

**Keywords:** disaster medicine, flood preparedness, primary health care

## Abstract

*Background*: Floods affect over 85 million people every year and are one of the deadliest types of natural disasters. The health effects of floods are partly due to a loss of access to health care. This loss can be limited with proper flood preparedness. Flood preparedness is especially needed at the primary health care (PHC) level. Flood preparedness assessments can be used to identify vulnerable facilities and help target efforts. The existing research on PHC flood preparedness is limited. We aimed to assess the flood preparedness of PHC facilities in a flood-prone province in central Vietnam. *Methods*: Based on flood experience, the PHC facilities in the province were grouped as “severe” (*n* = 23) or “non-severe” (*n* = 129). Assessments were conducted during monsoon season at five facilities from each group, using a pre-tested, semi-structured questionnaire. Data were checked against official records when possible. *Results*: Nine of the ten facilities had a flood plan and four received regular flood preparedness training. Six facilities reported insufficient preparedness support. Half of the facilities had additional funding available for flood preparedness, or in case of a flood. Flood preparedness training had been received by 21/28 (75%) of the staff at the facilities with severe flood experience, versus 15/25 (52%) of the staff at the non-severe experience facilities. *Conclusions*: Our results suggest that the assessed PHC facilities were not sufficiently prepared for the expected floods during monsoon season. PHC flood preparedness assessments could be used to identify vulnerable facilities and populations in flood-prone areas. More research is needed to further develop and test the validity and reliability of the questionnaire.

## 1. Introduction

Every year, floods affect over 85 million people globally [1]. Between 2012 and 2014, floods accounted for 42% of the nearly 40,000 natural disaster deaths, making floods the deadliest type of natural disaster during this period [2]. Death and physical injuries are direct health effects of floods. Indirect health effects include infectious diseases, malnutrition, and exacerbation of non-communicable diseases [3,4]. Indirect health effects result partly from the loss of access to health care. This loss may be due to damaged infrastructure such as roads, lack of medicines and equipment, or the destruction of health facilities. To limit the health effects of floods, attention has shifted from post-disaster management to pre-disaster planning [5], with an increasing emphasis on preparedness [6].

Primary health care (PHC) facilities are located in close proximity to the population and form an essential part of the health system following floods [7]. The role of PHC facilities is to maintain the provision of routine health services and cater for the excess burden of disease caused by the flood [8]. Preparing PHC facilities for floods is crucial to ensure an effective response. Flood preparedness can include situational action plans, or the maintenance and provision of relevant resources, such as power generators, vehicles, and drugs. In addition, the importance of regular training for flood situations, e.g., implementation of flood plans and evacuation plans should be underlined [9].

Flood preparedness assessments could help identify strengths and weaknesses at the PHC level. Assessments may be used to guide the implementation of measures that are adapted to the local PHC context, to ensure a better flood response. Previous assessments have found insufficient flood preparedness at PHC facilities in Asian flood-prone low- or middle-income countries [10,11,12,13]. Although some studies exist, research on PHC flood preparedness is scarce [5], and is especially needed in low- and middle-income countries with fragile health systems and limited resources [14]. The aim of this exploratory study was to assess flood preparedness among ten PHC facilities in a flood-prone province in central Vietnam.

## 2. Materials and Methods 

### 2.1. Study Setting

Vietnam is a lower-middle income country [15], and one of the most flood-prone countries in the world [16]. Between 2000 and 2012, the country experienced 32 floods that affected over 32 million people and caused more than 2100 deaths [17]. This study was conducted in the Thua Thien Hue province, located on the eastern coast of central Vietnam. The river networks in between the Truong Son Mountains and the South China Sea make the province prone to flash floods and riverine floods caused by tropical cyclones and heavy precipitation during monsoon season. The province has 152 PHC facilities that provide acute care and chronic disease management to a population of 1.1 million [18].

### 2.2. Assessment Questionnaire

The basic framework of the assessment questionnaire was created from literature identified during a search in September 2013. No validated means of assessment was identified during the search. Therefore, a semi-structured assessment questionnaire was constructed. Ten key components of PHC flood preparedness were extracted from the identified articles [10,11,19,20,21,22,23,24,25]. These components were divided into two groups: assessment by direct observations (facilities, equipment, and supplies) and assessment by key informant interviews (geographical data, climate history, human resources, training, planning, coordination, and funding). These assessment groups were combined with items from the World Health Organization (WHO) manual for community emergency preparedness [26], and the WHO checklist for the development of health care facility mass casualty management plans [27], to create a semi-structured assessment questionnaire (Supplement A). The aim was to cover all aspects of PHC flood preparedness, based on the WHO framework of six health system building blocks: (1) service delivery, (2) health workforce, (3) information, (4) medical products, vaccines, and technology, (5) financing, and (6) policy and governance [28].

The questionnaire was translated from English to Vietnamese by a person not directly involved in the research, and then back-translated to English by a member of the research team. Discrepancies in the Vietnamese version were corrected. A pilot test was performed at one PHC facility, using the Iarossi checklist [29], and changes were made to ensure clarity and relevance to flood preparedness, PHC facilities, and the context. The pilot test is not included in the results.

### 2.3. Study Sites 

To ensure the relevance and feasibility of the questionnaire for use in flood preparedness, PHC facilities with previous flood experiences were assessed. All PHC facilities of Thua Thien Hue province were categorized by their experience of flooding between 2008 and 2012, based on a review of official records and local expert advice. PHC facilities with “severe” (*n* = 23) and “non-severe” (*n* = 129) flood experience were identified. A flood was considered severe if the PHC facility was forced to close due to flooding or if one person or more in the uptake area of the facility had been rendered homeless by the event.

A sub-group of seven facilities was created in each group. In the group with severe experience, seven facilities were identified as the most affected, based on the number of people affected. In the group with non-severe experience, the facilities were listed alphabetically and seven facilities were selected using a random number table. Through expert advice, five facilities in each sub-group were selected by their proximity to rivers, mountains, and the coast (Table 1), with the aim of ensuring geographical diversity.

### 2.4. Data Collection

The PHC facilities were contacted beforehand and an appointment was made for the visit. No details on the planned assessments were given. The assessments were performed during monsoon season in October 2013. Since any day could be the start of a severe flood, the questionnaire was filled in according to the situation on the day of the assessment, disregarding improvement plans. Information on coverage area, previous flooding, the facilities, equipment, supplies, human resources, flood plans, and funding was recorded in the questionnaire. Data were checked against official records when possible. The quantity of the objects was not noted, only “available” or “unavailable”. Objects were marked as “available” if they were observed in the facility by the research team. Non-functional equipment was recorded as “unavailable”.

The two main researchers conducted the pilot test and all ten facility assessments. Key informant interviews were done with the head (*n* = 9) or the vice head (*n* = 1) of the facilities. Compensation was provided for loss of income, based on hourly wages.

### 2.5. Data Analysis

Data obtained from the interviews and direct observations were recorded on paper-copies of the assessment questionnaire and then entered into an Excel^®^ spreadsheet (Microsoft, Redmond, WA, USA). Descriptive analysis was performed: mean and median was used for summarizing numerical variables, and percentages for categorical variables.

### 2.6. Ethical Consideration 

This study was performed in accordance with the Declaration of Helsinki, and the protocol was approved by the ethical committee of Hue University of Medicine and Pharmacy, Vietnam prior to initiation (15 October 2013). Written informed consent was obtained from all participants and all participants were informed that they could refuse to participate or withdraw from the study at any time.

## 3. Results

The average assessment time was 69 minutes (range: 45–100). Nine of the 10 facilities had a flood plan and four received regular, routine training on flood preparedness. While all key informants considered their current preparedness guidelines to be sufficient, six reported insufficient preparedness support. None of the key informants reported satisfactory preparedness training and funding. Half of the facilities had additional funding available for flood preparedness, or in case of a flood. Flood preparedness training had been received by 21/28 (75%) of the staff at the facilities with severe flood experience, versus 15/25 (52%) of the staff at the non-severe experience facilities. While only two facilities coordinated with other primary health care centers (both in the severe group), all facilities reported coordination with a district or city health center (Table 2).

The availability of resources at the facilities varied (Table 3). Electricity, toilets, communication sets, pharmacies, wound management supplies, antibiotics, and hypertension treatment were available at all the facilities. Two of the ten facilities had a power generator, one had access to a car, and none had plaster of Paris for fracture management or hemoglobinometer. Other laboratory resources (glucometer, microscopy) were also lacking. No facilities had tetanus prophylaxis or insulin available.

## 4. Discussion

The results from this exploratory study suggest that the assessed PHC facilities were not sufficiently prepared for the expected floods during monsoon season. Based on the WHO model of six health system building blocks [28], the main flood preparedness deficiencies were found in relation to financing, medical products, vaccines and technology, and health workforce training. In the case of flooding, access to secondary care is often limited and PHC facilities need to be prepared to provide care for patients with traumatic injuries, and communicable and non-communicable diseases [30]. All facilities in this study lacked the necessary equipment and medicines to provide this care, such as plaster of Paris, tetanus prophylaxis, and insulin.

Proper PHC flood preparedness could reduce health effects and limit the number of non-seriously ill patients seeking secondary care. All the assessed facilities were able to coordinate with other district or city health centers. However, referral to another health care facility would be challenging, especially during a flood, given that only one PHC facility had a means of transport available. Although some roads were partially flooded at the time of our assessments, all the selected PHC facilities were accessible by motorbike. Each facility had an average catchment population of 9500 (Table 1), suggesting that flood preparedness could play a large and important role in the reduction of health effects in this context.

Only half of the facilities had separate funding available for flood preparedness or in case of a flood, and all facilities considered their flood preparedness funding insufficient. It appears that a lack of training was an important deficiency in the facilities we surveyed. These findings are in accordance with previous research from the same geographic region [12,13]. Initiatives to produce evidence-based disaster training exist for hospital health care workers [31]. Guidelines need to be developed for PHC facilities, like those assessed in this study. Through improved training of health care workers, the disaster preparedness of a facility may be strengthened [32]. Primarily, existing training and education mechanisms should be used [33]. The effectiveness of such training and exercises can be assessed by using qualitative and quantitative analysis [34]. If found insufficient, training should be offered regularly and be given at the facility level. The effects of such interventions need to be evaluated by repeated assessments, i.e., before and after implementation.

We believe this paper adds to the corpus of knowledge of flood preparedness by exemplifying an approach to performing rapid disaster preparedness assessments. The major practical contribution of the study is that it illustrates how a quick and relevant assessment tool can be used to identify vulnerable facilities. The next step is to decrease facility vulnerability. How that may be accomplished is beyond the scope of our study but may include training.

While this paper aims to describe the process and overall results of assessing PHC facility flood preparedness, certain methodological limitations should be taken into consideration. Firstly, the sample size was small and the facilities were purposively selected, and may not represent the general facilities or level of preparedness in this region. Secondly, the assessment questionnaire was created for the exploratory purpose of this study, and was not validated before use. The results could serve as a point of departure for further research on how to assess flood preparedness, identify vulnerable facilities, and prompt early action. The validity and reliability of the questionnaire need to be tested in order to determine if the questionnaire is accurate enough to evaluate different aspects of PHC flood preparedness. Finally, while steps were taken to cross-reference collected data, such as recent flooding events, with official records, the results are subject to some bias due to the recall method used in the key informant interviews. Strengths of the study include a novel approach to flood preparedness assessments by combining direct observations with short, semi-structured interviews.

## 5. Conclusions

We have documented our experience with assessing PHC flood preparedness using a self-developed, pre-tested, semi-structured assessment questionnaire. We found it feasible to perform a flood preparedness assessment in just over one hour. Although based on a non-validated assessment, our results indicate that the flood preparedness at the PHC facilities assessed in Thua Thien Hue province needs to be strengthened. PHC flood preparedness assessments could prove to be a key tool for strategic development by identifying vulnerable facilities in flood-prone areas. Vulnerable facilities should be provided with sufficient funding and offered relevant training. Future research to further develop and validate the questionnaire, as well as a systematic approach to assessment in a larger sample, including the community, could lead to a better understanding of flood preparedness in this context.

## Figures and Tables

**Table 1:** Coverage areas and history of recent flooding among severely and non-severely flooded primary health care facilities.

Coverage and Flooding History	Severe (*n* = 5)	Non-Severe (*n* = 5)	All (*n* = 10)
Coverage			
Total number of villages/citizen groups	54	73	127
Total catchment population	45,165	50,073	95,238
Average number of patients seen per month (range)	820 (350–1250)	458 (120–850)	639 (120–1250)
Experienced flooding, number of facilities			
2007	5	2 ^1^	7
2008	5	2 ^1^	7
2009	5	4	9
2010	5	3	8
2011	5	3	8
2012	3	2	5
2013	5	0	5
Flood-related deaths			
Total between 2007 and 2013	9	6	15

^1^ Response missing from two facilities.

**Table 2:** Human resources and the availability and sufficiency of flood preparedness training and funding among severely and non-severely flooded primary health care facilities, total *n* = 10.

Human Resources, Flood Preparedness, and Funding	Severe (*n* = 5)	Non-Severe (*n* = 5)	All (*n* = 10)
Human resources			
Doctors per facility, mean (range)	1.2 (1–2)	1.0 (1–1)	1.1 (1–2)
Assistant doctors ^1^ per facility, mean (range)	2.0 (2–2)	2.2 (1–3)	2.1 (1–3)
Nurses or midwives per facility, mean (range)	1.8 (1–3)	1.4 (1–2)	1.6 (1–3)
Facilities with staff trained in flood preparedness			
Doctors	3	3	6
Assistant doctors ^1^	4	2	6
Nurses or midwives	3	2	5
Facility has a flood plan	5	4	9
Routine flood preparedness training provided by government ornon-governmental organization	3	1	4
Separate funding available annually for flood preparedness or in case of a flood	5	0	5
Current flood preparedness training and funding is sufficient	0	0	0
Current flood preparedness guidelines are sufficient	5	5	10
Current flood preparedness support is sufficient	3	1	4
Facility coordinates with another primary health care facility	2	0	2
Facility coordinates with a district or city health center	5	5	10

^1^ Trained two years at medical college, compared to six years for medical doctors.

**Table 3:** Availability of observable resources at severely and non-severely flooded primary health care facilities.

Resources	Severe (*n* = 5)	Non-Severe (*n* = 5)	All (*n* = 10)
Facility-wide resources			
Electricity	5	5	10
Toilets	5	5	10
Pharmacy	5	5	10
Emergency food supply for staff	4	2	6
Emergency water supply for staff	5	2	7
Car for rescue and relief	1	0	1
Power generator	1	1	2
Communication set ^1^	5	5	10
Autoclave for sterilization	4	3	7
Wound and fracture management supplies			
Cleansing solution, dressing materials	5	5	10
Basic splints	5	4	9
Plaster of Paris	0	0	0
Laboratory resources			
Glucometer	0	2	2
Hemoglobinometer	0	0	0
Microscopy or rapid detection for malaria	0	1	1
Urinalysis	4	4	8
Drug supplies			
Oral analgesics	5	5	10
Local analgesics	4	3	7
Antibiotics for respiratory tract infection	5	5	10
Antibiotics for diarrheal disease	5	5	10
Tetanus prophylaxis	0	0	0
Malaria treatment	1	2	3
Treatment for hypertension	5	5	10
Treatment for asthma	5	5	10
Insulin	0	0	0
Diazepam	3	1	4

^1^ E-mail, telephone, or fax.

## Supplementary Materials

The following is available online at http://www.mdpi.com/1660-4601/15/12/2689/s1, Supplement A: Flood Preparedness Assessment Form.
