# Supplementary material for: Hope for the Best, Prepare for the Worst—An Assessment of Flood Preparedness at Primary Health Care Facilities in Central Vietnam
Source: Int J Environ Res Public Health. 2018 Nov 29;15(12):2689. doi: 10.3390/ijerph15122689 (PMC6313744; doi:10.3390/ijerph15122689)
Supplement: Supplementary file 1 [file ijerph-15-02689-s001.pdf]

FLOOD PREPAREDNESS ASSESSMENT FORM
**Factors influencing level of primary health care flood preparedness in THUA THIEN HUE PROVINCE**

Date:

Name of CHC:

Head of CHC:

Address:

Phone number:

A. General information

**1. Geographical data**	*Item*	*Comment*		
a. Straight line distance to the coast (km)				
b. Straight line distance to nearest main river or lagoon (km)				
c. Height above sea level (ground floor) (m)				
**2. Coverage area**	*Item*	*Comment*		
a. Number of villages				
b. Number of population				
c. Average number of patients seen per month (doctor/midwife/nurse, all visit types, incl. follow-up)				
**3. Climate history**	*CHC or covered area affected by flood (y/n)*	*No. of affected villages*	*No. of affected people, e.g. damaged house or health effect*	*No. of flood deaths*
2007				
2008				
2009				
2010				
2011				
2012				
2013				

B: Key informant interview

**4. Human resources**	*Number*	*I. Trained in flood preparedness*	*II. Trained in basic life support, incl. rescue breaths & chest compression.*	*III. Trained in advanced life support, incl. tracheal intubation & cardiac defibrillation*	*IV. Trained in emergency & trauma care*	*V. Trained in endemic disease management*
a. Doctors						
b. Assistant doctors						
c. Midwives						
d. Nurses						
e. Pharmacist						
f. Staff supervision (y/n)						

**5. Planning**	*Available/Unavailable*	*Comment*
a. CHC flood plan (i.e. routines, guidelines)		

C. General information

**6. Training, planning, funding & health effects**	*Comment*
a. What routine trainings are given by government or NGOs (I-V listed in question 4 or other trainings)?	
b. Is there any separate funding for flood preparedness annually? Any funding in case of flood?	
c. Is current training and funding sufficient?	
d. How and when are routines and guidelines received (e.g. yearly by fax)?	
e. What support is given in case of flood? By whom?	

f. Is current situation regarding guidelines and support sufficient?	
g. Is the CHC doing any community work to raise awareness regarding flood preparedness (e.g. disease prevention, ensure safe water quality)?	
h. What are the general health effects seen at the CHC following floods?	
i. What is the average change in patients seen after a flood (%)?	

D. Key informant interview

**7. Coordination**	*Available/Unavailable*	*Comment*
a. Other CHCs		
b. District/city health centre		
c. Provincial/central hospital		
**8. Additional information**		

E: Direct observations

**9. Facilities**	*Available/Unavailable*	*Comment*
a. flood safe construction (e.g. buildings on stilts)		
b. Water supply		
c. Electricity supply		
d. Toilets		
e. Outpatient facilities with roof while waiting		
f. Outpatient facilities with roof while receiving care		
g. In ward facilities		
h. Delivery room		
i. Pharmacy		

j. Medical record		
k. Emergency food stockpile for staff members		
l. Emergency water stockpile for staff members		
m. Car available for rescue and relief		
n. Generator set		
o. Communication system, i.e. e-mail or phone or fax		
**10. Equipment**	*Available/Unavailable*	*Comment*
a. Basic steam autoclave for sterilization		
**11. Supplies**	*Available/Unavailable*	*Comment*
Initial wound care		
a. Solution for wound cleansing, e.g. potable water or normal saline		
b. Simple dressing material		
Basic fracture management		
c. Basic splints		
d. Plaster of Paris (POP)		
Laboratory		
e. Glucose		
f. Haemocue or similar		
g. Rapid detection for malaria or microscopy		
h. Urine analysis to estimate the presence of sugar, red cells and white cells, e.g. urine dipstick		
Drug supply		
i. Oral analgesia		
j. Parenteral analgesia		
k. Local analgesia		
l. Antibiotics for respiratory tract infections		
m. Antibiotics for diarrhoeal disease		

n. Tetanus prophylaxis		
o. Malaria treatment		
p. Treatment for hypertension		
q. Treatment for asthma		
r. Insulin		
s. Diazepam		
**12. Additional information**		
